# Cytokines and Chemokines as Biomarkers of Future Asthma

**DOI:** 10.3389/fped.2019.00072

**Published:** 2019-03-19

**Authors:** Andrew Bush

**Affiliations:** Departments of Paediatrics and Paediatric Respiratory Medicine, Royal Brompton Harefield NHS Foundation Trust and Imperial College, London, United Kingdom

**Keywords:** eosinophilia, airway remodeling, airway hyper-responsiveness, bacterial infection, allergy

## Abstract

Antenatal and preschool factors are key in determining the progression to pre-school wheeze and eosinophilic school age asthma. The conventional view of eosinophilic asthma is that airway inflammation is the fundamental underlying abnormality, and airway inflammation and hyper-responsiveness are secondary; in fact, these three are parallel processes. Very early structural changes, independent of inflammation and infection, are associated with early airway hyper-responsiveness and later adverse respiratory outcomes. There is a bidirectional relationship between structural airway wall changes and airway inflammation, with airway contraction *per se* leading to the release of growth factors, and inflammatory pathways promoting airway remodeling. Early viral infection (and increasingly being appreciated, bacterial infection) is important in wheeze outcomes. There is evidence of abnormal immune function including cytokine release before the onset of viral infections. However, viral infections may also have prolonged effects on the host immune system, and the evidence for beneficial and adverse effects of viral infection is conflicting. In older children and adults, asthmatic epithelial cells show impaired interferon responses to viral infection, but only in the presence of uncontrolled type 2 inflammation, implying these are secondary phenomena. There are also compelling data relating the innate immune system to later asthma and atopy, and animal studies suggest that the effects of a high endotoxin, microbiologically diverse environment may be modulated via the epithelial alarmin IL-33. Whereas, previously only viral infection was thought to be important, early bacterial colonization of the upper airway is coming to the fore, associated with a mixed pattern of TH1/TH2/TH17 cytokine secretion, and adverse long term outcomes. Bacterial colonization is probably a marker of a subtle immune deficiency, rather than directly causal. The airway and gut microbiome critically impacts the development of Type 2 inflammatory responses. However, Type 2 inflammatory cytokines, which are critical both to progression from pre-school wheeze to eosinophilic asthma, and sustaining the eosinophilic asthmatic state, are not implicated in the very early development of the disease. Taken together, the evidence is that the earliest cytokine and chemokine signals will come from the study of bronchial epithelial cell function and their interactions with viruses and the microbiome.

## Introduction

The *Lancet* commission defined asthma as a clinical umbrella term, like arthritis, and anemia, comprising combinations of wheeze, chest tightness, breathlessness, and sometimes excessive cough (1). The term “asthma” is thus the start, not the finish of the diagnostic journey, and the next question is, “what sort of “asthma” are we considering”? For the purposes of this chapter I will consider the two commonest pediatric asthmas; (a) non-atopic episodic pre-school wheeze (characterized by fixed and variable airflow obstruction, no eosinophilic airway inflammation, but recurrent viral and bacterial infection) and (b) atopic allergic school age asthma (also fixed and variable airflow obstruction, but dominated by eosinophilic airway inflammation). It should be noted of course that although much non-atopic episodic wheeze remits by school age, this is not necessarily the case, and persistence into the teenage years, without progression to atopic wheeze, is well-described. The key questions are (1) how can we predict which babies will start to have acute, episodic wheeze, and (2) how do we prevent non-atopic pre-school wheeze progressing to atopic allergic school age asthma? What we want is objective biomarkers to define at risk infants, knowledge of the endotypes driving disease progression and treatment strategies to prevent this happening. Although these questions are the focus of much research activity, we do not know any of the answers, so any discussion as to whether measurements of cytokines and chemokines will be helpful will of necessity be speculative. Hence the purpose of this chapter is to indicate areas which may be a fruitful hunting ground, rather than to provide definitive answers.

A major part of the problem is the lack of data on the normal development of immune and epithelial function and the interaction with the evolving microbiome, in large measure due to the difficulty of obtaining relevant biological samples in normal children. Without knowledge of normal developmental pathways, and cytokine and chemokine expression, it is difficult to interpret disease states. This lack is currently being addressed (below).

## The Pathophysiology of Eosinophilic Asthma

The traditional model of school age and adult asthma is that airway inflammation drives both airway hyper-responsiveness (AHR) and airway remodeling. This simplistic view has been challenged by studies showing that neither AHR nor inflammation at baseline (2, 3) have any close correlation. Also, change in AHR and change in inflammation with treatment such as omalizumab (4) and infliximab (5), do not correlate. Furthermore, the evidence is that airway remodeling as conventionally described (increased reticular basement membrane thickening, increased airway smooth muscle, goblet cell hyperplasia, and increased bronchial circulation, for example) correlates with the extent and duration of inflammation (6, 7); the best evidence is that these are parallel processes. Thus, it would be a fundamental error to look for biomarkers of future asthma solely in inflammatory pathways. Indeed, as discussed below, Type 2 inflammation, although the hallmark of much school age and later asthma, is a late arrival on the airway scene.

## The First Steps: Episodic Viral Wheeze

The first signal of airway disease in virtually all babies is wheezing with a usually clinically diagnosed acute respiratory viral infection. Depending on the diagnostic fashion of the region, this may be labeled episodic viral wheeze (although strictly, bacteria may also have an important role to play, see below), bronchiolitis, or viral pneumonia. Many of these babies remit, but some go on to atopic allergic eosinophilic asthma in school age. Factors driving persistence of wheeze include severe acute attacks of wheeze and multiple aeroallergen sensitization (8, 9). The endotypes driving these wheezing phenotypes are unknown, and one can only speculate about any role of cytokines and chemokines in triggering the disease or leading to its progression. However, although endotypes are not known, there are data suggesting where there may be fruitful areas of enquiry. These include the role of antenatal structural changes in the airway, the innate immune system, the immune response to viral infection, and interactions with the evolving airway and gut microbiome, considered in more detail below. Finally, although traditionally acute wheeze is thought related to early respiratory virus infection, there is mounting evidence that bacterial infection may also be important, although the nature of the relationship between bacterial infection, viral infection and wheeze is still controversial.

### Is There a Role of Very Early Structural Airway Changes in the Pathophysiology of the Asthmas?

Numerous animal studies have demonstrated that adverse intra-uterine effects (usually nicotine, signaling through the α_7_ nicotinic acetylcholine receptor) alter the structure of the developing fetal lung, including increasing collagen deposition (10), up-regulating MUC5AC expression (11), airway lengthening and reduction in caliber (12), abnormal airway branching (13), and failure of alveolar development leading to early emphysema (14) and loss of the alveolar guy ropes which stabilize the airway (15). In one study (12), the post-natal readout was AHR, *in the absence of any inflammation or airway infection*. Three groups measured AHR in new-born babies (16–18), before any significant viral infection, and presumptively before the onset of eosinophilic inflammation. All three showed that AHR was associated with long term adverse respiratory outcomes, including a greater risk of being given a diagnosis of asthma. Indeed, in one study, AHR was a stronger predictor of asthma age seven than airway obstruction at birth (18).

How might structural changes *per se* lead to AHR? There are at least two potential mechanisms which are not mutually exclusive. The first is altered airway dimensions, with airway narrowing, and elongation leading to increased resistance (which has been shown to occur in animal models, above), resulting in a bigger signal than in a structurally normal airway if resistance is increased still further by another stimulus. The second is the loss of the airway stabilizing effect of the delicate alveolar tethering points which hold open the airways by the phenomenon of interdependence, which has also been demonstrated in animals and humans (15, 19). Neither seems likely to have a cytokine or chemokine based signal, or at least, not one that is known.

Human studies are also informative. In a follow up study of infants with really severe wheeze who underwent airway biopsy at 30 months of age (20), increased airway smooth muscle, but not eosinophilic inflammation or reticular basement membrane thickening, was predictive of a school age asthma diagnosis (21). Another follow up study also failed to implicate eosinophilic inflammation as a marker of future asthma risk; there were insufficient biopsies for smooth muscle to be assessed, but reticular basement membrane thickness was predictive of a subsequent asthma diagnosis (22, 23).

Is it biologically plausible that structural changes signaling through AHR, could be the primary initiating abnormality leading to asthma, or at least the early loss of lung function which is one of the hallmarks of the disease? A study in adult asthma patients compared the effects of bronchoconstrictor challenges with methacholine (which is not pro-inflammatory) and house dust mite (which causes eosinophilic airway inflammation as well as bronchoconstriction) (24). Methacholine challenge led to structural changes in the airway without causing any airway inflammation. These included reticular basement membrane thickening, increased epithelial expression of Transforming growth factor (TGF) β and Ki67 and increased epithelial PAS positivity. The effects of methacholine were blocked by salbutamol, which prevented bronchoconstriction. It has been hypothesized that phasic contraction and relaxation of the developing fetal airway is an important drive to the release of growth factors important for lung development (25–27). In lung slices, bronchoconstriction-induced remodeling can be demonstrated and abrogated by PDE4 inhibitors and anti-muscarinic agents (28). So I hypothesize that early abnormal AHR leading to structural airway changes via the release of cytokines, chemokines and growth factors is a key initiator of airway remodeling, and searching for biomarkers in families of growth factors, including TGF-β and insulin-like growth factor, may be fruitful (29).

There is likely a subsequent bi-directional interaction between the airway wall and inflammation, with links between type 2 inflammation in particular, and structural airway wall changes. One example, which again draws attention to the epithelium, is the alarmin IL-33-epithelial growth factor (EGF) pathway. As a result of allergen sensing, IL-33 is released and signals through lineage negative innate lymphoid cells through the ST-2 receptor. These cells also bind amphiregulin, a bi-functional growth factor which may induce cell proliferation or differentiation. A number of studies have demonstrated the importance of this axis in children with asthma. There is a correlation between IL-33 positive cells in the submucosa and reticular basement membrane thickness in children with severe therapy resistant asthma (30). Innate lineage negative cells (ILC), in this case Type 2 are increased in bronchoalveolar lavage (BAL) from children with severe asthma, compared with controls being investigated for recurrent infection (31). Increased sputum amphiregulin and EGF in pediatric asthma correlate with AHR and sputum eosinophilia (32, 33). The expression of EGF receptor is increased in asthmatic bronchial epithelium (34). However, it seems likely that these pathways are more important in the propagation of asthma, rather than its inception, given that allergic sensitization and Type 2 inflammation are not early features of wheeze (below).

### The Immune Responses to Respiratory Viruses and Subsequent Wheeze

The question of whether particular viral infections “cause” subsequent eosinophilic asthma in children who otherwise would not have the disease is discussed in detail below. This section focuses on the early factors leading to acute wheeze with respiratory viral infections.

Firstly, pre-existing structural airway changes are important determinants of viral induced respiratory illness. Pre-existing airway obstruction was demonstrated in the Perth cohort in babies hospitalized for bronchiolitis, and this tracked into mid-childhood (35). It is certainly biologically plausible that wheezing is more likely to be detected clinically if there is inflammation and oedema in a pre-narrowed airway. But there is also evidence of altered ante-natal immunological responses in those who subsequently wheeze with viral infections. The COAST study recruited 285 newborns and measured peripheral blood mononuclear cell (PBMC) cytokine secretion to phytohaemagglutinen (PHA) stimulation at birth (cord blood) and 1 year of age (36). They documented respiratory viral infections by culture and PCR. Those with multiple viral infections had less marked interferon (IFN)-γ responses in cord blood PBMC to PHA stimulation, and less decline over a year in such IFNγ responses. The effects of maternal smoking in pregnancy (a known association of more severe respiratory viral infection in the baby) have also been studied. Cord blood PBMCs from infants of mothers who smoked in pregnancy showed increased proliferation to stimulation by HDM (37). The Perth group (38) showed that the babies of mothers who smoked in pregnancy had reduced responses of toll-like receptors (TLR) to their ligands, specifically reduced TLR2 responses to IL-6, IL-10, and TNF-α and reduced TLR3 and 4 responses to TNF-α. The TLR9 pathway showed reduced IL6 responses, but an increased response to stimulation with IFN-γ. Thus, there are clear cut data that there are immunological abnormalities before any direct exposure to viral infection or respiratory treatments in babies who go on to wheeze with viral colds. However, these are very descriptive studies, and it is unclear how these abnormalities relate to future respiratory outcomes, or whether in fact they are merely markers of a more fundamental process. However, more sophisticated cord blood studies may allow delineation of at-risk populations and also endotypes of disease. Certainly cord blood cytokine and chemokine PBMC studies, combined with transcriptomics and other more sophisticated molecular tests, look to be a promising area for predicting the risk of asthma.

We now know that the epithelium, far from being a mere passive barrier, has many sensory functions as the first site of interaction with micro-organisms and pollutants, and is the source of numerous cytokines and growth factors. Initial studies in adults with asthma have shown that there are impaired anti-viral mucosal responses, with deficient release of type I interferon-β and type III interferon-λ in response to rhinovirus stimulation (39, 40), and this was confirmed in children with severe asthma (41, 42). What is not known is whether this is a primary abnormality or secondary to repeated viral infections or iatrogenic secondary to treatment. However, the fact that IFN responses are normal in well-controlled asthma (43) suggests that the abnormalities previously described relate to the inhibitory effect of Type 2 inflammation (44, 45). This is the likely mechanism whereby inhaled corticosteroids (ICS) reduce the frequency of asthma attacks in atopic, school age children (46). Hence abnormalities in the epithelial IFN pathways seem an unlikely cause of progression from pre-school wheeze to asthma, but are more likely a later phenomenon related to Type 2 inflammation.

There are limited data on airway epithelial cell function at birth and later wheeze outcomes. In one study, nasal epithelial cells were harvested in newborns as a surrogate for the lower airway (47). They were followed up by parental questionnaire to determine wheeze outcomes. Ninety-one of 139 questionnaires were returned, of whom there were 16 children who had ever wheezed and 11 who had recently wheezed. When compared to those with no recent wheeze, supernatants from cultured neonatal airway epithelial cells taken at birth from the children with recent wheeze had reduced IL-8 release after stimulation with culture medium alone, TNF-α/IL-1β and lipopolysaccharide. The cells also exhibited reduced release of IL-6, GM-CSF, and ICAM-1 on stimulation with TNF-α/IL-1β and reduced release of ICAM-1 and RANTES after house dust mite stimulation. Unfortunately, IFN responses to viral stimulation were not studied. Although this study has weaknesses (small numbers, reliance on parental report of wheeze, no wheeze phenotyping, the inevitable requirement to assume that upper airway epithelial cells are a good surrogate for lower), it nonetheless provides important proof of concept that epithelial cell function is abnormal at birth in children who go on to wheeze. A subsequent study (48) in older children (up to age 16 years) showed that, at baseline, nasal epithelial cells from children with a history of wheeze produced significantly less IL-8, IL-6, MCP-1, and G-CSF, but not VEGF, RANTES, MMP-9, or TIMP-1, than healthy controls. After stimulation with IL-1β and TNFα, cells from children with current wheeze produced significantly less IL-8, IL-6, and MCP-1 than control children, but release of G-CSF, VEGF, MMP-9, and TIMP-1 was similar. Of note, wheeze but not atopy was the only predictor of cytokine release. Taken together, these lines of evidence suggest that abnormal epithelial cytokine release is important in wheeze, and a search for endotypes to asthma in the epithelium may be fruitful. Furthermore, at least in established wheeze and asthma, we know that the epithelium is damaged, with evidence of increased epithelial loss (49), increased shedding of epithelial cells in BAL (50), increased EGFR expression in bronchial biopsies (51), and a reduced wound-healing ability despite elevation in plasminogen activator inhibitor-1 (52), again suggesting that epithelial function may hold the key to predicting future risk of asthma.

Although there are compelling data of pre-existing immune abnormalities in children who go on to wheeze with respiratory viruses, there may be subsequent interactions between viral infections, and the host immune responses, and disease pathogenesis. Theoretically, such interactions could be beneficial, reducing the risk of future viral infection and wheeze, or adverse, and the evidence is conflicting. If these endotypes could be discovered, in particular epithelial cytokine responses to viral infections could be a marker to understand the pathways to progression or remission of pre-school wheeze.

In favor of a beneficial effect of early viral infections is one interpretation of David Strachan's original observation (53), confirmed many times in different contexts and by different groups, that the greater the number of older siblings, the less likely the child was to have atopic disease (“Hygiene hypothesis”). This could be because the youngest child acquires more early respiratory viral infections from the siblings. In support of this, there is a protective effect for firstborn infants who are placed early in a child care facility (54). In this regard, there is at least some biological plausibility that early inflammation may be protective from murine skin studies (55). Skin barrier function is maintained by epithelial stem cells. In this report, the investigators demonstrated that there was a prolonged memory to early acute inflammation which resulted in the stem cells restoring barrier function after subsequent tissue injury more efficiently, independent of macrophages or T cells. The mechanism is likely the maintenance of chromosomal accessibility at key stress response genes which were activated by the initial stimulus, allowing genes governed by these domains to be transcribed more rapidly in response to subsequent challenge. The absence of AIM2, caspase-1 and IL-1β prevents this protective effect.

However, there is also evidence of a harmful effect of early viral infections on longer term immunological responses. A large randomized controlled trial of RSV prophylaxis with palivizumab showed reduction in viral wheeze even outside the RSV season (56), implying RSV infection had more than transient acute effects. There are a number of potential cytokine based mechanisms whereby RSV infection may impact future Type 2 inflammation. For example, infection of a neonatal but not adult murine model with RSV induced rapid IL-33 expression and an increase in ILC2 numbers; this was not observed in adult mice (57). Treatment with an anti- IL-33 monoclonal abrogated AHR, Type 2 inflammation, airway eosinophilia, and mucus hypersecretion. IL-33 receptor knockout mice were also protected. Interestingly, treatment of adult mice with IL-33 returned the effects of RSV infection to those of infancy; how adult mice suppressed a harmful IL-33 response is not known. Infants hospitalized with RSV had elevated IL-33 and IL-13 in nasal aspirates, levels of which declined during recovery. This gives a possible mechanism linking IL-33 and ILC2s to future asthma after RSV infection. Physiologically, lung epithelial cells inhibit T-cell proliferation; this braking effect is lost upon stimulation with RSV or poly (I:C), another potential pathway whereby RSV may switch on Type 2 inflammation (58, 59).

An important birth cohort study measured viral infection and immune responses in nasal lavage, and related these to the specific virus identified and wheeze outcomes over the following 2 years (60). Briefly to summarize a huge amount of data, they showed that in RSV but not RV infected infants, an immune pattern characterized by down-regulation of IFN pathways, and upregulated Type-2 and -17 pathways were associated with recurrent wheeze in the succeeding 2 years. Growth factors and chemokines were central to RV immune responses. However, it should be noted that in the COAST study, the strongest associations between early viral infection and subsequent asthma were for RV, and not RSV infection (61, 62). Hence perhaps of more relevance was a report from the Manchester, UK Birth Cohort relating RV cytokine responses of peripheral blood mononuclear cells, admittedly perhaps a less relevant cell type (63). They measured 28 cytokines after stimulation with RV-16 in 307 children aged 11 years, using machine learning to relate patterns of cytokine responses to clinical outcomes, using longitudinal models. There were six clusters of children based on their rhinovirus-16 responses, which were differentiated by the expression of four cytokine/chemokine groups (IFN-related, proinflammatory, Type 2 chemokine, and regulatory). They found that early-onset troublesome asthma with early-life sensitization, later-onset milder allergic asthma, and protection from wheeze were each associated with different patterns of immune responses to RV. Of course, it would have been of more interest to know whether these cytokine responses preceded viral infections, and whether they were present in early life. Nonetheless, taken together, these studies suggest that the early cytokine responses to respiratory viral infection may be important in the later development of both early episodic viral wheeze and later eosinophilic, atopic asthma. It is also worth noting that RV and RSV infection is virtually universal in infancy, and the vast majority of those afflicted do *not* develop any later airway disease (64); study of protective pathways may open novel therapeutic avenues.

### The Role of Innate Immunity in Subsequent Wheeze

One of the striking challenges in understanding asthma pathophysiology is the repeatedly replicated finding of a low prevalence of asthma and atopy in babies born into farming families and brought up on the farm (65, 66). This has been related to environmental microbial diversity and endotoxin levels (66). A recent study has provided powerful evidence that the innate immune system may modulate these effects, and thus measuring innate immune signature cytokines could be a useful biomarker of asthma risk (67). The Amish and Hutterite peoples, who are genetically almost identical, immigrated to the USA from Europe more than 200 years ago. The Amish retained very traditional farming methods, and as expected, had a much lower prevalence of asthma and allergic sensitization than the Hutterites, who have switched to modern methods (5.2 vs. 21.3% and 7.2 vs. 33.3%, in Amsih and Hutterites for asthma and allergic disease, respectively). The Amish environment had much higher dust endotoxin levels as expected. Increased environmental bacterial and fungal diversity also appears to be beneficial. Compared to the Hutterites, Amish children had increased peripheral blood neutrophils, and reduced eosinophils; monocyte counts were similar. Amish neutrophils expressed lower levels of the chemokine receptor CXCR4 and the adhesion molecules CD11b and CD11c, probably because they had recently exited the bone marrow. Amish but not Hutterite monocytes showed a suppressive phenotype, with lower levels of human leukocyte antigen DR (HLA-DR) and higher levels of the inhibitory molecule immunoglobulin-like transcript 3 (ILT3). There was no difference in regulatory T-cell (Treg) numbers. After innate (lipopolysaccharide) stimulation of peripheral blood leucocytes, 22 cytokines [Interleukin (IL)−17,−33,−31,−25,−27,−4,−5,−22,−2,−15,−10,−9,−13, 1β,−6,−28A,−23,−12p70, and IFN-γ, TNFα, MUP3a, and GM-CSF] were detectable, with the median levels of each being lower in the Amish children than in the Hutterite children. After adaptive immune pathway stimulation (combined anti-CD3 and anti-CD28 antibodies), there was no difference between the populations. The gene expression profiles of peripheral blood mononuclear cells were also strikingly different, with 1,449 genes differentially up-regulated in the Amish children and 1,360 genes up-regulated in the cells of Hutterite children. These differentially expressed genes were organized into 15 co-expression modules. The most significant network contained 43 genes and was associated with Amish and Hutterite status, and thus peripheral blood neutrophil and eosinophil counts. Eighteen of these genes were overexpressed in the Amish and were clustered in a network with hubs tumor necrosis factor (TNF) and interferon regulatory factor 7 (IRF7), both proteins being key to the innate immune response to microbials. These results were taken forward in the conventional murine allergic airway disease model. House dust extracts from Amish and Hutterite homes were administered intranasally, and bronchoalveolar lavage (BAL) obtained, and AHR measured. Hutterite dust exacerbated ovalbumin induced BAL eosinophilia and AHR. However, Amish dust extracts inhibited ovalbumin-induced AHR, BAL eosinophilia, and elevation of serum ovalbumin-specific IgE. All measured BAL cytokines were suppressed by Amish dust inhalation. MyD88 and Trif are two molecules critical in multiple innate immune-signaling pathways, and mice deficient in these molecules failed to show any Amish dust induced protection. These results were taken forward in a further murine experimental study (68). Both recombinant IL-33 (rIL-33) and allergen (house dust mite or *Alternaria alternata*) exposure from day 3 of life led to significantly increased pulmonary IL-13^+^CD4^+^ T cells, without which AHR did not develop. House dust mite exposure of neonatal IL-33^−^/^−^ mice was still sufficient to cause AHR. However, neonatal mice in whom IL-13^+^CD4^+^ T cells had been knocked out which were exposed to allergen from day 3 of life did not develop AHR despite persistent pulmonary eosinophilia, elevated IL-33 levels, and IL-13^+^ ILCs. Importantly, neonatal mice did not develop AHR when given inhaled *Acinetobacter lwoffii* (found in the environmental of cattle farms, and known to protect from childhood asthma) was co-administered with HDM. *A. lwoffii* prevented pulmonary IL-13^+^CD4^+^ T cells increasing in numbers, but had no effect on IL-13^+^ ILCs and IL-33. The importance of these data for this chapter are two-fold; first, they provide a novel pathway whereby environmental bacteria may abrogate asthma risk; and secondly, they suggest that the alarmins, although important in established pediatric asthma, may not be as relevant to its inception, or at least, may be one of a number of important factors.

An opportunistic study also implicated innate immunity; 64 children diagnosed with autoimmune neutropenia in the first year of life were compared with 415 controls matched for age, gender, indoor passive smoke exposure (69). There was no difference in the prevalences of asthma or eczema in first degree relatives. One of the neutropenic children developed wheeze, compared with 9.9% of the controls. Interestingly, 3 neutropenic patients developed school age wheeze, but only after resolution of neutropenia, suggesting that without neutrophils, early pre-school wheeze does not develop, and the infant appears to be protected against subsequent asthma.

Taken together, these data suggest an important role in innate immune function and signaling pathways in the early pathophysiology of asthma. These pathways also show potential for therapeutic intervention.

### What Are the Roles of Early Bacterial Infection?

Traditionally, bacterial infection has not been thought to have a role in the pathophysiology of asthma or acute wheeze. However, a landmark study (70) showed that children with positive hypopharyngeal cultures for *Streptococcus pneumoniae, Haemophilus influenzae, Moraxella catarrhalis*, and *Staphylococcus aureus* in very early infancy were more likely to have persistent wheeze and acute severe attacks of wheeze, and elevated blood eosinophil count and total but not specific IgE. Reversible airflow obstruction and an asthma diagnosis aged 5 years was also commoner in those colonized as babies. Clearly it could be argued either that bacterial colonization was causal, or that it was a marker of a low grade immune deficit which predisposed both to neonatal infection and also independently, to subsequent asthma. In a subsequent study (71), the mucosal immune response to these colonizing bacteria was determined by collecting nasal lining fluid. There was no clearly understandable immune signature. *M. catharralis* and *H. influenza* were associated with a mixed TH1/TH2/TH17 picture with high levels of IL-1β, TNF-α, and MIP-1β, and a subsequent increased risk of asthma aged 7 years (72, 73). *St. aureus* was associated with a TH17 profile and elevated IL-17; of note, there was no signal with *S. pneumoniae* colonization. Subsequent work on outcomes in colonized children has shown that they have increased troublesome non-specific respiratory symptoms (74), an increased risk of bronchiolitis and pneumonia (75), and interestingly, have low grade systemic inflammation as shown by an elevated hs-CRP (76). Furthermore, children given a diagnosis of asthma at age 7 years were shown to have increased levels of IL-5 and IL-13 when their peripheral blood mononuclear cells harvested at age 6 months were stimulated with *H. influenzae, M. catarrhalis*, and *S. pneumoniae*, and also increased IL-17 and IL-10 when stimulated by *H. influenzae* and *M. catarrhalis*, but not *S. pneumoniae* (72, 73). The COPSAC data have also been confirmed in a different cohort (76), in this case implicating pneumococcal colonization.

Of note, there are important interactions between bacteria and RSV in preschool respiratory illness. The RSV G glycoprotein promotes adherence of *S. pneumoniae* to respiratory epithelium, which associates with increased severity of wheeze attacks (77). Conversely, co-infection with RSV and either Haemophilus influenza or *S. pneumoniae* leads to increased expression of IL-6 and IL-8 in nasal secretions, and increased expression of signaling pathways associated with macrophage and neutrophil signaling (78). Further evidence of the importance of the interactions between RSV and the airway microbiome, this time apparently signaling through metabolic pathways, came from a multicenter study of hospitalized infants (79). The investigators showed that a panel of 25 metabolites, most strongly correlated with the relative abundance of *S. Pneumoniae*, predicted disease severity as shown by the need for positive pressure ventilation. Of these pathways, sphingolipid metabolites showed the most significant correlation. Whether these metabolites were causal of disease severity, or a marker of another, cytokine, or other factor driven pathway which was responsible for the more severe clinical course, could not be determined.

There is at least some mechanistic data as to how bacterial infection could lead to Type 2 inflammation (80). In a nasal polyp model, *S. aureus* increased expression of the epithelial alarmins IL-33, and TSLP, and the Type 2 cytokines IL-5, and IL-13. TSLP and IL-33 receptor expression, which was predominantly on CD3^+^ T cells, was also increased. However, *S. aureus* led to the release only of TSLP from inferior turbinate tissue. *S aureus* infection of BEAS-2B epithelial cells led to the release of IL-33 and TSLP together with activation of NF-κB (nuclear factor κB) pathways, and this was inhibited by specific TLR-2 antagonists (IL-25 was not measured). Hence one could propose a model whereby a subtle mucosal or more likely systemic immune defect led to an inability to clear bacteria from mucosal surfaces, and these bacteria subsequently activate alarmins, and drive a TH2 response via TLR-2 to lead to subsequent atopic, allergic asthma. However, and not mutually exclusively, this hypothetical defect could lead to asthma by leading to a dysregulated response to allergens.

Taken together, these data suggest that an aberrant immunological response is the primary event underlying early bacterial colonization, which event independently leads to asthma. This view is consistent with the COPSAC data, which showed that the *number* of respiratory infections in the first year of life is the most important predictor of subsequent airway disease, but there is no association with a particular viral or bacterial pathogen (81). If this is the case, then predictive cytokine biomarkers for asthma could be discovered by *in vitro* stimulation of nasal epithelial cells (which are easily obtained by brush biopsy even in young infants) or peripheral blood mononuclear cells.

### The Airway and Gut Microbiome and Inflammatory Cytokine Responses

There is far less information on the role of the developing gut microbiome, merely tantalizing hints of important interactions but no unifying hypothesis. In a murine model, animals raised in germ-free conditions had exaggerated Type 2 inflammatory responses to subsequent house dust mite challenge compared to control mice (82). There were no effects on IL-10 or IFN positive cells. Other findings included lower IgA in the BAL of germ-free mice, increased numbers of basophils, but decreased alveolar macrophages and plasmacytoid dendritic cells; Tregs were unaffected. These changes were reversed by re-constituting the mice with specific pathogen free microbes. The developmental importance of the airway microbiome was studied in another murine model (83). Newborn mice exposed to house dust mite developed marked type 2 airway inflammation and AHR despite the presence of Tregs. In the first 14 post-natal days, the nature and quantity of the bacterial load in the lungs changed, with increased bacterial phyla and a change from *Gammaproteobacteria* and *Firmicutes* being the predominant organisms toward Bacteroidetes. These changes were associated with decreased aeroallergen responsiveness and the emergence of a Helios(−) Treg cell subset that required interaction with programmed death ligand 1 (PD-L1) for development. The newborn pattern of responses to allergens could be extended into adult life by preventing microbial colonization or blockade of PD-L1 in the first 2 weeks of life, whereas adoptive transfer of Tregs from adult mice to neonates before aeroallergen exposure abrogated Type 2 inflammation and AHR. Hence the reduction in susceptibility to allergen challenge required the development of a normal microbiota.

The importance of the early microbiota is suggested by studies relating mode of delivery to subsequent atopic disease. Vaginal, but not cesarean delivery results in the infant being exposed to and colonized by the maternal vaginal and fecal flora (84), and is associated with reduced risk of atopy and food allergy (85–87). This is not necessarily an argument for home delivery, but infants of atopic parents delivered vaginally at home had reduced risks of eczema (odds ratio 0.52, 95% confidence intervals 0.35–0.77) and asthma (0.47, 0.29–0.77) (88). *S. pneumoniae* (89), *E. coli* (90), and *Helicobacter* (91) have all been shown to modulate airway immune and inflammatory responses.

The infant gut microbiota, as well as that of the airway, is important. *Clostridium difficile* isolation from stools at 1 month of age was associated with wheeze and eczema at 6–7 years of age (88). In another murine study (92), the importance of the gut-lung axis signaling through the interactions between diet and gut bacteria to alter the ratio of *Firmicutes* to *Bacteroidetes*. The gut microbiota metabolized dietary fiber, with the result that the concentration of circulating short-chain fatty acids (SCFAs) increased, leading to protection against allergic inflammation in the lung; a low fiber diet had the opposite effects. SCFA propionate treatment resulted in enhanced bone marrow generation of macrophage and dendritic cell (DC) precursors. The end result in the lungs was an increased DC population which were highly phagocytic and also impaired the ability to promote type 2 cell effector function, signaling through G protein-coupled receptor 41 (GPR41, also known as free fatty acid receptor 3 or FFAR3).

Finally, in terms of established adult and school age asthma, there is clear evidence of an abnormal airway microbiome (93), although as ever, it is impossible to dissect out the effects of recurrent infection, wheeze attacks and their treatment from those of disease. However, this is at least strong supportive evidence of the importance of microbiota-associated pathways. As yet, more detail is needed before we can determine whether the signaling pathways are cytokine based, or via the release of bacterial metabolites, or some other pathway(s).

### Is There a Role for Type 2 Inflammation in the Initiation of Allergic Atopic Asthma?

A number of lines of evidence show that the onset of Type 2 inflammation is a late phenomenon, as part of established atopic allergic asthma, and therefore looking for markers of future asthma in this pathway is highly unlikely to be fruitful.

Although it is well-known that low-dose ICS are superb treatment for atopic allergic asthma in school age children and adults, three well-constructed randomized controlled trials showed that early initiation of ICS either as regular therapy (94, 95) or intermittently during acute pre-school wheeze (96) are not disease-modifying and do not prevent progression to school age, atopic allergic asthma. This also ties in with histopathological and physiological data. Two cross-sectional studies of young children with wheeze sufficiently severe to justify referral to a specialist Children's Hospital for invasive investigation (20), the first in infants and the second at pre-school age shed light on the evolution of airway histology from normal to the typical inflamed, remodeled airway of allergic school age asthma. The study in infants (median age 12 months) investigated in Helsinki Children's Hospital (20), showed that even atopic children with documented reversible airflow obstruction had no evidence of airway inflammation or remodeling. In the second study (20), infants with confirmed wheeze at a median age 30 months already had evidence of reticular basement membrane thickening and eosinophilic airway inflammation. In terms of physiology, infants who go on to have persistent wheeze lose lung function, never to regain it, during the time period that remodeling and eosinophilic inflammation develop.

A novel study, which challenges preconceptions about early wheeze reported on a group of infants with symptoms of sufficient severity to merit a clinically indicated bronchoscopy (97). The infants were categorized into episodic and multiple trigger wheeze clinically; however there was no relationship between clinical symptoms and any allergic or inflammatory marker. Interestingly, analysis of the microbiota showed two distinct clusters: a *M. catarrhalis* positive, neutrophilic group, and a mixed culture, lymphocyte and macrophage predominant group. More work is needed, but these data again suggest that infection and the host response may be key in initiating airways disease, and therein are cytokine and chemokine predictors of asthma to be found.

Given that numerous studies in asthmatic patients treated with high dose steroids have shown that only minimal reversal of established remodeling is feasible (97, 98), it seems highly unlikely that Type 2 cytokines will allow us to predict the onset of atopic allergic asthma with enough lead time to allow a preventive intervention. There is a single study suggesting that (99) plasma cytokines (TARC, MDC, IP-10, not selected a priori from a big panel which was measured) measured at age 3 years may be able to predict asthma at age 6; however, this study had no second validation cohort, and must be considered at best hypothesis generating.

## The Progression From Episodic Viral Wheeze to Eosinophilic Asthma

There is really very little evidence about the pathways whereby infants with episodic viral wheeze either go into remission or progress to eosinophilic, school age asthma. That acute attacks of wheeze and multiple early aeroallergen sensitization are important associated factors is clear. Whether either causal, or merely a marker of an independent process is unclear. There are tantalizing hints that perhaps specific viral and bacterial infections may be important, and evidence that very early life factors are significant (above). However, it is clear that once changes of remodeling and Type 2 inflammation have set in, it is too late to reverse the march to eosinophilic school age asthma. We do not know how the pathways to disease arise, and currently, have no means of reducing risk.

In order to address this, the Breathing Together, Wellcome Trust funded consortium [http://breathingtogether.co.uk/] is attempting to dissect out the very earliest evens in pre-school wheeze (100). The group has recruited a birth cohort of 1,000 babies. Nasal epithelial cells as a surrogate for bronchial have been harvested and cultured within 2 weeks of birth. Nasal, pharyngeal, and throat swabs have been collected for microbiome studies, and a blood spots taken for transcriptomics. The babies are followed prospectively with repeated sampling of nasal cells, blood and microbiome in the first year of life, and at 1 and 3 years. A monthly on-line video-questionnaire about respiratory symptoms is filled in by the parents. The aim is to relate the initial samples and the developmental changes in cellular and immune function, and the microbiome to wheeze outcomes. Additionally, in order further to understand the pathobiology of end stage disease and try to relate it to the developmentally evolving pathways, namely pre-school wheeze and eosinophilic asthma phenotypes, three other groups will be studied. The first is children undergoing routine pediatric surgery, from whom blind lower airway lavage and brushings will be taken, as well as nasal brushings, microbiome studies, and peripheral blood; and severe pre-school wheezers and severe, therapy-resistant asthmatics who are undergoing a clinically indicated bronchoscopy. The same samples will be taken in these last two groups, the only difference being the lower airway samples will be taken under direct vision rather than blindly. A further use of the samples from these latter groups is the comparison of upper and lower airway samples, so we can determine how good a reflection of the relevant pathways the nasal cells from the birth cohort actually are. There is also a bi-directional interaction with the STELAR asthma e-lab (101), which houses data including genetics on more than 14,000 children from the British birth cohorts. Any pathway looking promising in the blood and cellular studies will have a genetic signal sought in STELAR. Conversely, any genetic signal will be validated using cellular studies, progressing to animal work if necessary. It is hoped that endotypes will be dissected out with this prospective approach. It is also intended to work with the EAGLE consortium on further genetic studies.

## Summary and Conclusions: Where From Here?

This chapter has reviewed potential avenues where studying cytokines and growth factors may enable us to determine risk factors for the development of asthma. It is very clear that the pathways from asymptomatic baby at birth to intermittent viral wheeze to remission or progression to eosinophilic asthma are unknown, and we do not have any empirical interventions which work. We know that early airflow obstruction persists lifelong, with a greatly increased risk of later COPD (102, 103). If we are to intervene, it will need to be before the child goes to school, and this means that the very early years are where the answers must be found and interventions applied. For this author, focussing on epithelial function, the interactions of epithelial cells with viral and bacterial infection, and epithelial cytokine and chemokine production, is the most likely fruitful field of study. Allergic sensitization may well be important in propagating eosinophilic airway disease, but Type 2 inflammation is a late-comer to the asthmas, and likely, its onset means that the chance for disease-modifying interventions has been lost forever. So it seems unlikely that early initiation of monoclonals such as mepolizumab directed against TH-2 high asthma will be disease-modifying, although this should be tested at least initially in a suitable animal model (104). Monoclonals directed against the alarmins, or perhaps other components of the immune system, may be the answer. It is intriguing to speculate that asthma may be an infectious disease after all (105). Some of us are old enough to remember the derision with which the suggestion that spirochetes caused duodenal ulcer was received. Now antibiotic treatment of *Helicobacter pylori* cures them. Perhaps the wheel will turn full circle and our professional children will cure asthma with antibiotics after all.

## Author Contributions

The author confirms being the sole contributor of this work and has approved it for publication.

### Conflict of Interest Statement

The author declares that the research was conducted in the absence of any commercial or financial relationships that could be construed as a potential conflict of interest.
